# Perceived Speed of Compound Stimuli Is Moderated by Component Contrast, Not Overall Pattern Contrast

**DOI:** 10.1177/2041669516674959

**Published:** 2016-10-26

**Authors:** Kevin R. Brooks, Peter Thompson

**Affiliations:** Department of Psychology, Macquarie University, Sydney, NSW, Australia; Perception in Action Research Centre (PARC), Faculty of Human Sciences, Macquarie University, Sydney, NSW, Australia; Department of Psychology, University of York, York, UK

**Keywords:** speed perception, contrast, spatial frequency, motion perception

## Abstract

The perception of speed is susceptible to manipulations of image contrast, both for simple sine wave and more complex stimuli, such that low-contrast patterns generally appear slower than their high-contrast equivalents. It is not known whether the crucial factor is the contrast of the underlying Fourier components or the contrast of the overall complex pattern. Here, two experiments investigate this issue using compound gratings, comprising two vertical sine wave stimuli with equal contrast, but a 3:1 spatial frequency ratio. Component gratings were summed in “peaks add” and in “peaks subtract” phase, creating conditions with either (a) identical component contrasts, despite differences in overall pattern contrast or (b) differences in component contrasts despite identical overall pattern contrast. Experiment 1 demonstrated that the perceived speed is determined by the contrast of the components regardless of relative phase and hence of overall pattern contrast. Experiment 2 replicated this result while eliminating potential explanations based on differences in spatial frequency content. Along with previous compound grating and plaid studies, the data support a two-stage velocity estimation process involving the derivation of separate speed signals for each Fourier component, followed by integration of these signals across spatial scales.

## Introduction

The perception of motion is automatic and seemingly effortless, yet despite a substantial volume of work over the last 30 years (see Burr & Thompson, 2011; Nishida, 2011 for recent reviews), many of the finer points of the underlying processes remain obscure. In particular, the mechanisms responsible for estimating stimulus speed have proved elusive. While speed perception can be remarkably precise under some circumstances (McKee, Silverman, & Nakayama, 1986), its accuracy is often compromised by changes of basic stimulus properties such as contrast. Under most circumstances, when the contrast of a stimulus is reduced, it appears to move at a slower velocity. This effect has been shown both for simple sine wave stimuli (Campbell & Maffei, 1981; Hawken, Gegenfurtner, & Tang, 1994; Johnston, Benton, & Morgan, 1999; Stone & Thompson, 1992; Thompson, 1976, 1982; Thompson, Brooks, & Hammett, 2006; Thompson & Stone, 1997), and more complex stimuli that include many Fourier components with different spatial frequencies, orientations, phases, and contrasts (Blakemore & Snowden, 1999; Brooks, 2001; Brooks, Morris, & Thompson, 2011; Brooks & Rafat, 2015; Horswill & Plooy, 2008; Snowden, Stimpson, & Ruddle, 1998). While this effect is robust to many experimental variations such as psychophysical procedure and stimulus type, it is unclear whether its magnitude depends on the contrast of the underlying components or the contrast of the overall pattern.

**Figure 1.**
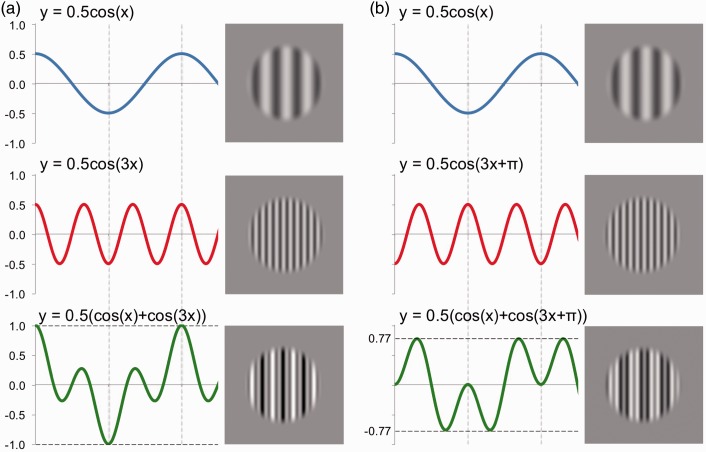
The summation of vertical sine wave gratings (top and middle) to form compound 1D stimuli (bottom) with different relative phase relationships. (a) Condition 1: peaks add. Peaks of the low SF stimulus (top) coincide with peaks of the higher SF grating (middle), while troughs also coincide. The resulting compound (bottom) has an overall pattern contrast equal to the sum of the two component contrasts. (b) Condition 2: peaks subtract. Peaks of the low SF stimulus coincide with troughs of the higher SF grating, and vice versa. Although component contrasts are the same as in (a), overall pattern contrast is only 77% of the sum of component contrasts.

For any natural image, the overall pattern contrast will correlate highly with the contrast of its Fourier components. However, there are situations in which the two can be dissociated. For example, when two sine waves with equal contrasts but a spatial frequency (SF) ratio of 3:1 are summed, the overall pattern contrast can vary depending on their relative phase. When the two are summed in “peaks add” phase, the contrast of the overall pattern is equal to the sum of the two component contrasts (Figure 1(a)). However, when the two are summed in “peaks subtract” phase, the contrast of the overall pattern reaches only 77% of this value (Figure 1(b)). This dissociation allows us to predict different patterns of results depending on whether perceived speed is dependent on component contrast or overall pattern contrast. If component contrast is crucial, the two compound stimuli in Figure 1(a) and (b) should appear to translate at the same speed. However, if overall pattern contrast moderates perceived speed, then the stimulus created in peaks add relative phase (Figure 1(a)) should appear faster than the “peaks subtract” stimulus (Figure 1(b)).

We conducted two experiments to establish the effects of component or overall pattern contrast using the stimuli described in Figure 1. In the first experiment, all compound stimuli were matched in speed to a simple, one-component sine wave grating, while in the second experiment compound stimuli with different phase relations were matched directly to each other.

## Experiment 1

### Methods

#### Subjects

Data were collected from 13 subjects, including author K. B.—the only non-naïve participant. All had normal or corrected-to-normal vision and were aged 18 to 50.

#### Design

This experiment measured the point of subjective equality (PSE) for a 1 c/deg sine wave grating compared with compound grating stimuli comprising two vertical sine wave gratings. The contrasts of these two sine wave components were always equal but could have one of three levels (independent variable #1: component contrast). Components could be presented with a phase relationship where the peaks either add or subtract (independent variable #2: relative phase). In half of the conditions, the compound grating served as the (variable) “test” stimulus, and the simple sine wave was the fixed “standard” stimulus, while the converse was the case in the remaining conditions (independent variable #3: standard/test configuration). All conditions were repeated at two different objective velocities, 1 and 4 deg/s, (independent variable #4: speed) to create an overall 3 × 2 × 2 × 2 design.

#### Apparatus and stimuli

Stimuli were displayed using a Sony Trinitron G520/Dell P1130 CRT monitor with a spatial resolution of 1,344 × 1,008, running at a frame rate of 120 Hz. The monitor was connected to a G5 Power Mac, housing an ATI Radeon HD 4870 graphics card, providing 10-bit grey level precision. Stimuli were programmed and generated through MATLAB, using the psychophysics toolbox (Brainard, 1997; Pelli, 1997). The screen subtended 7.6 × 5.7° from the 3 m viewing distance. Responses were made via a two-button mouse. The mean luminance of the linearized screen was 47 cd/m^2^, and all tests took place in a darkened laboratory.

Stimuli were superimposed on a mean luminance background in a circular aperture with a raised cosine profile and a 4° diameter, the outermost 0.5° being contrast-modulated to blend into the background. A small, high contrast central target served as a fixation point. Simple grating stimuli involved vertical cosine waves of 1 c/deg and a Michelson contrast of 1.0. Compound stimuli involved the sum of two vertical cosine gratings, one at 1 c/deg and the other at 3 c/deg, each with contrasts of 0.5, 0.385, or 0.296. It should be noted that neighboring values have a ratio of 1:0.77. The phase relationship between the two components was arranged such that their peaks add or subtract (i.e., differences in initial phase of 0 or π radians, respectively). Components always moved at the same velocity, maintaining their phase relationship throughout. Stimuli moved rightward or leftward in separate trials. As statistical differences involving direction were not theoretically relevant, PSE data for leftward and rightward conditions were averaged within each condition.

#### Procedure

In each trial, a standard and a test stimulus were presented sequentially (arranged in random order), centred on the fixation point, following which the subject was asked to indicate which stimulus (the first or the second) appeared to translate at a higher speed. The first trial was initiated by a button press. After 1000 ms, the two stimuli appeared, separated by an inter-stimulus interval (ISI) of 500 ms. Following the subject’s response, the next trial was initiated automatically following an inter-trial interval (ITI) of 1000 ms. During the ISI and ITI periods, a blank screen at mean luminance was displayed along with the fixation point. While the duration of the standard stimulus was fixed at 400 ms, the test could have one of five durations (300, 350, 400, 450, or 500 ms) selected at random, to discourage a strategy of simply comparing extents of displacement between the two intervals.

Trials were run in sets of randomly interleaved 1-up-1-down staircases, one for each experimental condition. In any given staircase, the speed of the standard stimulus was fixed while the speed of the test was manipulated on a trial-by-trial basis. Each staircase began with a test stimulus presented at a randomly determined speed either faster or slower than the standard speed, and featured variations of test speed in steps that began at 40% of the standard speed, halving in size every reversal until they reached the minimum step size of 5%. Each staircase terminated after nine reversals, with the final six reversals averaged as an estimate of the PSE. This occurred, on average, after 21 trials. During a single testing session, subjects were tested in four blocks of trials, administered in random order, with short rest periods in between. Each block used a set combination of speed and standard/test configuration. Within each block, 12 randomly interleaved staircases involved factorial combinations of component contrast, relative phase, and direction. Given the combination of data across direction, each PSE is calculated from trials involving 18 reversals, or approximately 42 total responses.

#### Analysis and predictions

The use of two levels of standard/test configuration, where both compound and simple gratings serve as test and as standard, allows us two opportunities to assess their relative perceived speed. If the compound stimulus should appear to have a different speed to the simple sine wave, this would result in a PSE higher than the objective standard speed in one condition, and a lower PSE in the other. Whatever the cause of the difference in perceived speed, this predicts patterns of results that are equal and opposite for the two levels of standard/test configuration. As such, we can combine the two conditions by expressing the test speed at the PSE as a proportion of the standard speed, and taking the reciprocal of the data in the Test Compound conditions to allow combination with the Standard Compound condition. Expressed as a *speed match* percentage, all data now represent the perceived speed of a compound stimulus, relative to a simple sine wave. A 3 × 2 × 2 × 2 ANOVA showed no statistically significant effects involving the standard/test configuration variable, legitimizing the combination.

**Figure 2.**
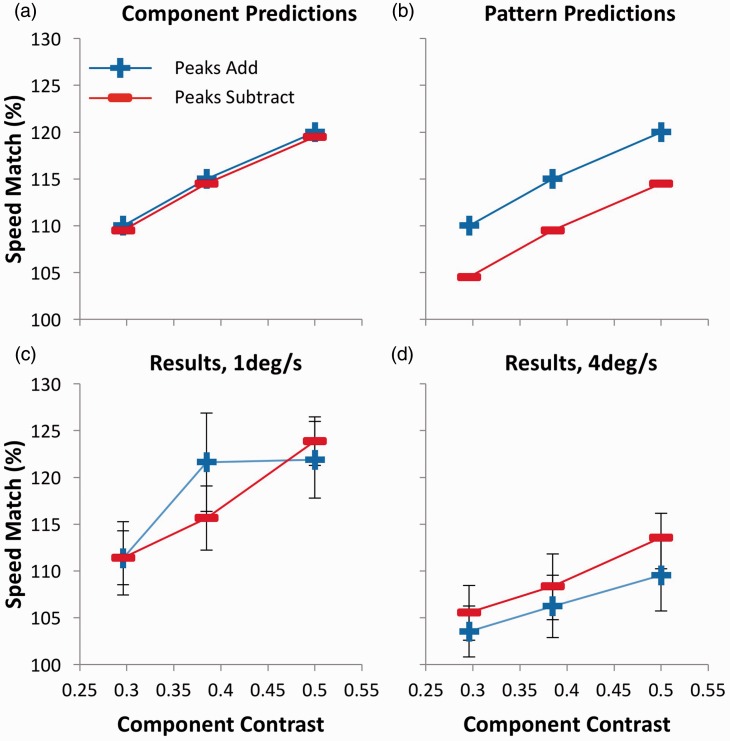
Predictions and results for Experiment 1, plotted in terms of component contrast. While (a) represents the predictions of a system where perceived speed is determined by component contrast, (b) represents the predictions of overall pattern contrast. (c) Results for 1 deg/s. (d) Results for 4 deg/s. Error bars represent ± 1 SEM.

Regardless of whether the perceived speed of compound gratings is determined by the contrast of components or of the overall pattern, a significant main effect of component contrast is expected, given that within each level of relative phase, overall pattern contrast increases as component contrast increases. The two possibilities—that perceived speed is determined either by component contrast or by overall pattern contrast—can be distinguished by the presence or absence of a significant main effect of relative phase. If perceived speed is determined by overall pattern contrast (Figure 2(a)), we should expect significance, as PSEs should be higher for peaks add compared with peaks subtract stimuli. However, if component contrast determines perceived speed, then this effect should not be significant (Figure 2(b)).

### Results and Discussion

PSEs for all conditions, plotted as a function of component contrast, are shown in Figure 2(c) and (d) for 1 and 4 deg/s, respectively. Consider the results for 1 deg/s. The key finding is that, while speed match values are higher when component contrast is higher, there is no difference between the peaks add and peaks subtract conditions. These observations were confirmed in formal statistical tests (2 × 2 × 3 ANOVA) (see Appendix A). A statistically significant main effect of component contrast was evident, *F*(2,24) = 13.752; *p* = <.0005; $\eta_{p}^{2}$^ ^= .534, as expected. Although a statistically significant main effect of speed, *F*(1, 12) = 10.741; *p* = .007; $\eta_{p}^{2}$^ ^= .472, showed that percent speed match values were higher at 1 deg/s, there were no interactions between speed and any other factor, confirming that the general pattern of results was similar at both speeds. No other statistically significant effects were found. Of specific relevance to this investigation, there was no effect of relative phase, *F*(1, 12) = 0.216; *p* = .650. These results are consistent with the idea that perceived speed is determined by component contrast rather than overall pattern contrast.

**Figure 3.**
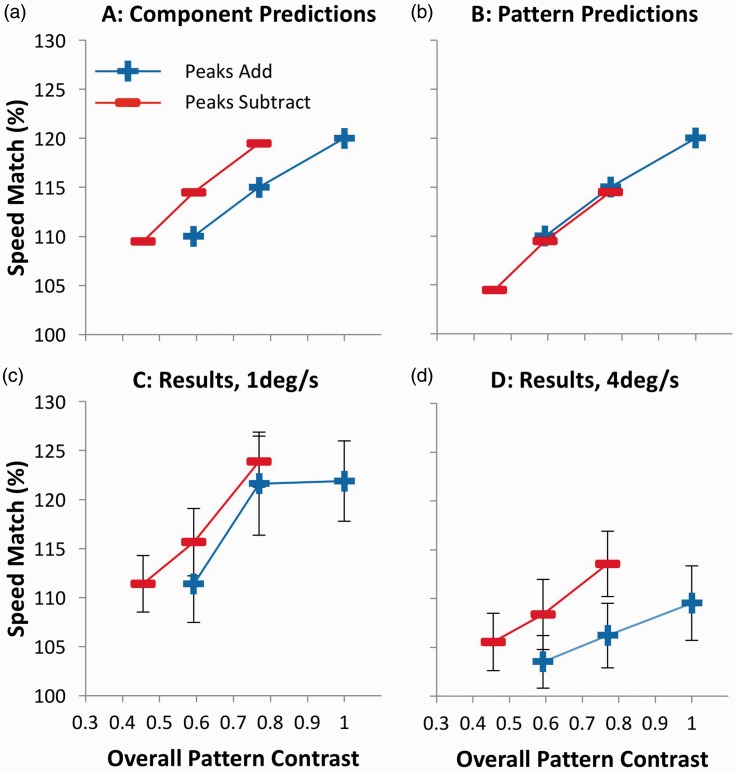
Predictions and results for Experiment 1, plotted in terms of overall pattern contrast. While (a) represents the predictions of a system where perceived speed is determined by component contrast, (b) represents the predictions of overall pattern contrast. (c) Results for 1 deg/s and (d) 4 deg/s. Error bars represent ± 1 SEM.

Although this experiment was principally designed for 2 × 2 × 3 analysis, the choice of three-component contrasts that differed with a ratio of 1:0.77—the same ratio that describes the relative amplitudes of a peaks add and a peaks subtract compound made from the same components—was no accident. This allows us to replot the data in terms of overall pattern contrast instead of component contrast. These data are shown in Figure 3, which demonstrates the alignment of the two lower contrast conditions when peaks add with the two higher contrast conditions when peaks subtract. Note that when overall pattern contrast is equal for a peaks add and a peaks subtract stimulus, the peaks subtract component contrasts are necessarily higher than those for peaks add stimuli. This allows us to perform a 2 × 2 × 2 ANOVA on the replotted data, with the independent variable of pattern contrast (two levels) replacing component contrast (three levels) in the previous analysis (see Appendix B). If component contrast determines perceived speed, a main effect of relative phase is predicted, as PSEs should be higher for peaks subtract compared with peaks add stimuli (Figure 3(a)). However, if overall pattern contrast is the determinant of perceived speed, then this effect should not be significant (Figure 3(b)). This effect was significant, *F*(1,12) = 9.652; *p* = .009; $\eta_{p}^{2}$^ ^= .446, providing positive evidence that component contrast, not overall pattern contrast, determines perceived speed.^1^

The data demonstrate that relative phase has no influence on perceived speed when stimuli are equated in terms of component contrast but show a clear effect when they are equated in terms of pattern contrast, at least for the stimuli used here. Although consistent with the contention that it is component contrast that influences perceived speed, the data cannot be accounted for by the proposal that overall pattern contrast is the crucial factor. However, this interpretation should be treated with a degree of caution, given that the predictions of the component contrast model relies in part on a null result for the original data set. Furthermore, positive evidence for the effect is only found when analysing the results as a function of pattern contrast (Figure 3) in a reduced data set, with two conditions excluded. In addition, complications are caused by the differences in spatial content of the standard and test stimuli in each trial. While the compound stimulus always contained two SFs (1 and 3 c/deg), the simple grating contained only one (1 c/deg). Previous studies have shown that for stimuli similar to those used here, SF affects perceived speed and that this relationship is moderated by contrast, with a more pronounced effect at higher contrasts (Brooks et al., 2011). As such, Experiment 1 served as a preliminary test of the essential hypotheses and provided evidence of consistency across stimulus speed. To eliminate any potential artifacts based on differences in SF, we ran a second experiment in which all stimuli were identical in spatial content. In addition, by careful design, we ensured that conclusions would not be based on an absence of an effect or on an effect in a reduced data set, with each model predicting a different combination of one significant difference and one null result.

## Experiment 2

### Methods

All methodological details were identical to Experiment 1, except in the following regards. A total of 12 subjects, including author K. B., between the ages of 18 and 40 were tested. Stimuli involved compound gratings only, with every standard and test comprising both 1 c/deg and 3 c/deg components. In each of the four conditions, one stimulus (either standard or test) was in peaks add phase with the other in peaks subtract phase, (see Table 1). In Conditions 1 and 2, the components had equal contrast (0.5) while their overall pattern contrasts differed (1.0 and 0.77). In Conditions 3 and 4, the component contrasts differ (0.385 and 0.5) while the overall pattern contrasts were the same (0.77). Thus, the factorial combination of independent variable #1: relative phase of the standard stimulus (peaks add/peaks subtract) and independent variable #2: match type (component or pattern contrast) produced a 2 × 2 within subjects design.

**Table 1.** Details of Standard and Test Stimulus Parameters for All Conditions in Experiment 2.

No.	Match type	Standard			Test		
		Relative phase	Component contrasts	Overall pattern contrast	Relative phase	Component contrasts	Overall pattern contrast
1	Component	Peaks add	0.5	1	Peaks subtract	0.5	0.77
2	Component	Peaks subtract	0.5	0.77	Peaks add	0.5	1
3	Pattern	Peaks add	0.385	0.77	Peaks subtract	0.5	0.77
4	Pattern	Peaks subtract	0.5	0.77	Peaks add	0.385	0.77

**Figure 4.**
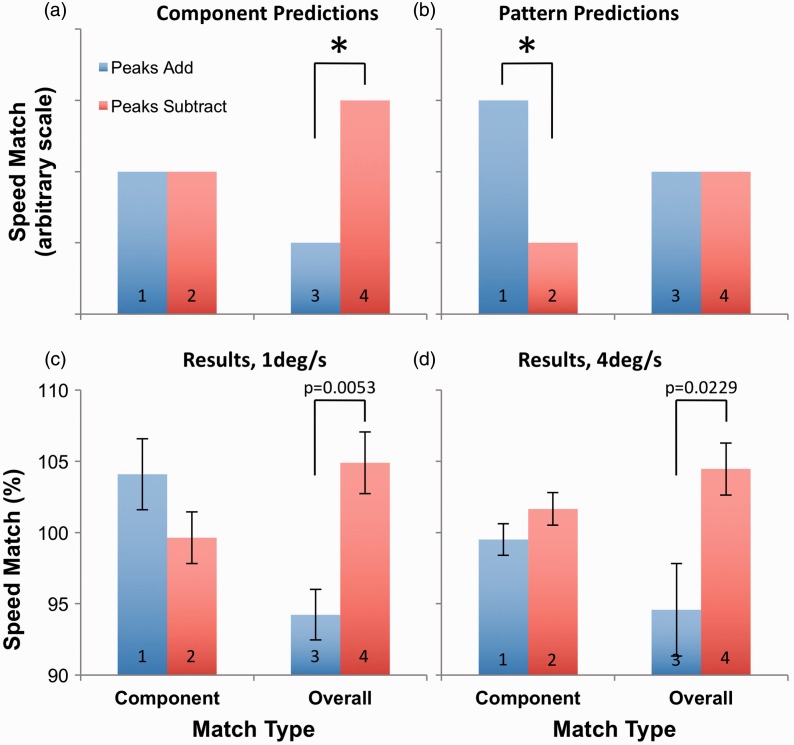
Predictions and results for Experiment 2. While (a) represents the predictions of a system wherein perceived speed is determined by component contrast, while (b) represents predictions based on the overall pattern contrast. (c) Results for 1 deg/s. (d) Results for 4 deg/s. Error bars represent ± 1 SEM.

As in Experiment 1, contrasting predictions can be made based on whether component or overall pattern contrast influences perceived speed. These are depicted in Figure 4(a) and (b). If perceived speed is dependent on component contrast, PSEs should be similar when component contrasts match (Conditions 1 and 2). However, when overall pattern contrasts match, the PSE should be lower when the standard stimulus’ peaks add (Condition 3), and higher when they subtract (Condition 4), as in Figure 4(a). Conversely, if perceived speed is dependent on overall pattern contrast, similar PSEs should be found when stimuli match in this respect (Conditions 3 and 4). When component contrasts match, the PSE should be higher when the standard stimulus’ peaks add (Condition 1), and lower when they subtract (Condition 2), see Figure 4(b).

### Results and Discussion

Results for Experiment 2 appeared to follow the same pattern for 1 and 4 deg/s stimuli (see Figure 4(c) and (d), respectively). A 2 × 2 × 2 ANOVA confirmed the generality of results across stimulus speed, showing no main effects or significant interactions that involved the independent variable of speed. However, there was a significant main effect of relative phase, *F*(1,11) = 8.159; *p* = .016; $\eta_{p}^{2}$^ ^= 0.426, and a significant interaction between relative phase and match type, *F*(1,11) = 7.292; *p* = .021, $\eta_{p}^{2}$^ ^= 0.399, as expected. The component match data at 1 deg/s (Figure 4(c)) may appear similar to the pattern predictions (Figure 4(b)); however, PSEs for conditions with peaks add and peaks subtract relative phase did not differ significantly at either speed (two-tailed *t* tests, 1 deg/s: *t*(11) = 1.546, *p* = .150; 4 deg/s: *t*(11) = −1.208, *p* = .253). Furthermore, when overall pattern contrasts matched, a higher PSE resulted when standard stimulus components were combined in peaks subtract phase compared with the condition in which the standard was peaks add (two-tailed *t* tests 1 deg/s: *t*(11) = −3.075, *p* = .011, *d* = 1.561; 4 deg/s: *t*(11) = −2.251, *p* = .046, *d* = 1.085). In each case, the effect size substantially exceeds Cohen’s (1988) convention for a large effect (*d* = .80).

This pattern of results indicates that the contrast of component gratings is the crucial variable, regardless of the relative phase in which they are combined and hence their overall pattern contrast. Furthermore, as the stimuli in Experiment 2 differ only in terms of the relative phase of their components, we can be confident that this experiment has isolated the contributions of component contrast and overall pattern contrast without the potential confound caused by differences in SF content.

## General Discussion

This study provides evidence that the perception of stimulus speed is mediated by the contrast of the Fourier components, rather than being determined by the overall pattern contrast, at least for the iso-oriented stimuli used here. From Experiment 1, the evidence comes through two key observations: (a) the lack of the predicted effect of relative phase when data are plotted in terms of their component contrast and (b) significant influence of relative phase when stimuli are plotted in terms of their overall pattern contrast. From Experiment 2, evidence comes in the form of equivalence of perceived speed for stimuli whose component contrasts matched while pattern contrasts differ, along with a simultaneous difference in perceived speed for compound gratings whose overall pattern contrasts match yet their component contrasts differ.

While observations from Experiment 1 are suggestive, caution should be used in interpreting null results and positive results that are based on a reduced data set. In addition, caveats remain in terms of the lack of equivalence of spatial content, given that compound stimuli were always compared with a simple sine wave grating. However, observations from Experiment 2—the presence of statistically significant differences in perceived speed alongside such null results in a specific pattern—is far more convincing in statistical terms, while eliminating any possible influence of SF. Combining all observations, significance was present and absent in the pattern predicted by the influence of component contrast, with overall pattern contrast proving inconsequential. This is true for both of the speed ranges tested and was shown in both experiments.

While many previous studies have investigated motion perception using compound stimuli formed from two-component sine wave gratings, only a handful of studies have involved iso-oriented gratings such as ours (Brooks et al., 2011; Priebe, Castellano, & Lisberger, 2003; Priebe, Lisberger, & Movshon, 2006; Smith & Edgar, 1991). The vast majority of studies have instead used gratings with different orientations combined to form a “plaid” pattern. For these stimuli, research investigated the properties of plaid components that would promote a percept of coherent motion of the unified pattern (rather than a percept of independent gratings sliding over each other in transparent motion), and the determinants of perceived pattern direction. Adelson and Movshon (1982) demonstrated that plaids tend to cohere when their contrasts and spatial frequencies are similar. These findings led them to suggest a two-stage model of motion perception, wherein the properties of each individual component were first encoded by early mechanisms before the true stimulus velocity could be computed at a higher level of processing. Consistent with this suggestion, direction-selective “component” neurons in V1 were shown to respond most vigorously when the plaid included components drifting in the neuron’s preferred direction, while “pattern” neurons in MT responded most vigorously when the unified plaid pattern (not the components) drifted in the cell’s preferred direction (Movshon, Adelson, Gizzi, & Newsome, 1985). Furthermore, stimulus manipulations that alter the perceived speed of the components (presumed to be processed at the earlier stage), such as those caused by SF (Smith & Edgar, 1991), by adaptation (Derrington & Suero, 1991), or by changes of contrast (Stone, Watson & Mulligan, 1990), have been shown to produce changes of the perceived direction of the plaid pattern.

Of particular relevance to the current study, the perception of plaid speed also appears to be consistent with a two-stage model of velocity computation. Even when the plaid is coherent and the motion of components is not perceptually accessible (Welch, 1989; Welch & Bowne, 1990), plaid speed discrimination thresholds are lowest when components move at speeds that are optimally discriminable, rather than when the features of the overall pattern move at optimally discriminable rates.

Although little research has looked at the issue of coherence in iso-oriented compound gratings such as ours, simple observation confirms that while contrast and SF do not appear to play a substantial role, similar speed is a prerequisite for coherence. The issue of perceived speed for compound gratings has been investigated by Smith and Edgar (1991), who suggested a two-stage process wherein the perceived speed of each component is encoded first, before a combination process establishes the perceived speed of the pattern. The results for many conditions were consistent with a simple averaging process, although some more complex stimulus conditions demanded a non-linear process to account for the pattern of speed misperceptions. Similar results were reported by Brooks et al. (2011), with the average of the components’ perceived velocities proving a relatively good predictor of perceived speed for many compound stimuli. Meanwhile, neurophysiological studies on speed tuning have shown some similarities to the aforementioned direction tuning for plaids (Movshon et al., 1985). While V1 and MT neurons will respond both to plaids and to their isolated components moving in the appropriate direction, their speed tuning properties can be quite different. That is, the preferred speed of V1 neurons for compound gratings may be predicted by a simple linear sum of its responses to each isolated component, but the same cannot be said for MT neurons. These higher level units show more complex response properties wherein the preferred speed for compound stimuli is not well predicted by responses to the components (Priebe et al., 2006). Instead, the activity of these units tends to be less susceptible to changes of SF (Perrone & Thiele, 2001; Priebe et al., 2003). Furthermore, unlike V1 cells, these units show narrower speed tuning bandwidths when stimulated by complex stimuli rather than simple gratings (Priebe et al., 2006). These results, along with other models of speed perception (Perrone, 2005, 2006; Perrone & Thiele, 2002) suggest that, as for the computation of plaid direction, V1 signals are forwarded to MT, where a non-linear interaction between the spatial frequencies present in the stimulus allows enhancements in speed perception accuracy and precision (Priebe et al., 2006).

Our results are consistent with the two-stage model of velocity computation for complex stimuli. In this model, a reduction of component contrast would affect the responses of neurons in the first stage. Such effects would be expected regardless of the phase of each grating, or of the relative phase relationship between the two. These responses, signalling a lower-than-veridical speed would then be sent to MT, whose neurons would inherit the reduced speed signal. Conversely, the current pattern of results is inconsistent with a single stage speed perception process, wherein the velocity of the compound, along with its overall pattern contrast, would be encoded without decomposition into components.

The finding that component contrast rather than overall pattern contrast mediates perceived speed allows additional confidence in the results of previous studies of perceived speed in compound gratings where the phase relationship between components was not always controlled in the manner used in this investigation (Brooks et al., 2011; Priebe et al., 2003, 2006; Smith & Edgar, 1991). According to the current study, relative phase makes no difference. Although our results stress the importance of Fourier components in the estimation of stimulus speed, we need not discard the results of experiments that specify the contrast of their images in terms of the overall pattern (e.g., Blakemore & Snowden, 1999; Brooks & Rafat, 2015; Horswill & Plooy, 2008; Krekelberg, van Wezel, & Albright, 2006). In natural images, the plethora of SFs and the intricate pattern of phase relationships means that in practice, peaks and troughs do not reliably add or subtract in a consistent way, ensuring a high correlation between overall pattern contrast and component contrast and hence the same predictions.

Despite the introspective impressions of the observers in this experiment, and of humans in daily life, we conclude that humans do not judge the speed of complex stimuli as a whole but instead implicitly combine the independent perceived speed contributions of each underlying Fourier component. How the speeds of these components are integrated has yet to be established. While Smith and Edgar’s (1991) suggestion that we simply average the two provides a decent approximation, it fails for certain stimuli. In particular, complex stimuli are less susceptible to the effects of SF on perceived speed, compared with isolated sine wave gratings, while high-contrast stimuli are generally more susceptible than their low-contrast equivalents. This causes discrepancies between the predictions of a simple averaging process and the actual perceived speed of compounds (Brooks et al., 2011). Hence, a more detailed model is required that is capable of accounting for the complex pattern of perceived speeds that results from the combination of simple Fourier stimuli.

## Notes

1 In addition, the main effects of pattern contrast, *F*(1,12) = 15.522; *p* = .002; ηp^2 ^= .564, and of speed, *F*(1,12) = 10.227; *p* = .008; ηp^2 ^= .460, were confirmed, as expected. No other statistically significant effects were present.
